# SNP rs2243828 in MPO associated with myeloperoxidase level and atrial fibrillation risk in Chinese Han population

**DOI:** 10.1111/jcmm.15644

**Published:** 2020-07-16

**Authors:** Pengyun Wang, Mian Cheng, Pengxia Wang, Liang Xiong, Yali Zeng, Xin Tu, Rongfeng Zhang, Yunlong Xia, Gang Wu, Qing Wang, Xiang Cheng, Chengqi Xu

**Affiliations:** ^1^ Department of Clinical Laboratory Liyuan Hospital Tongji Medical College Huazhong University of Science and Technology Wuhan China; ^2^ Department of Geriatrics Tongji Hospital Tongji Medical College Huazhong University of Science and Technology Wuhan China; ^3^ Key Laboratory of Molecular Biophysics of the Ministry of Education College of Life Science and Technology Center for Human Genome Research and Cardio‐X Institute Huazhong University of Science and Technology Wuhan China; ^4^ Department of Cardiology First Affiliated Hospital of Dalian Medical University Dalian China; ^5^ Department of Cardiology Renmin Hospital of Wuhan University Wuhan China; ^6^ Center for Cardiovascular Genetics Department of Molecular Cardiology Lerner Research Institute Cleveland Clinic OH USA; ^7^ Department of Molecular Medicine CCLCM Cleveland OH USA; ^8^ Department of Genetics and Genome Sciences Case Western Reserve University Cleveland OH USA; ^9^ Department of Cardiology Union Hospital Tongji Medical College Huazhong University of Science and Technology Wuhan China

**Keywords:** atrial fibrillation, genetic association, inflammation, myeloperoxidase

## Abstract

Previous studies shown that myeloperoxidase (MPO) level is higher in patients with atrial fibrillation (AF); however, no genetic evidence between *MPO* and AF risk in human population was observed. Therefore, the present study was aimed to investigate the association between rs2243828, a variant in promoter region of *MPO* and the risk of AF in Chinese GeneID population. The results demonstrated that the minor G allele of rs2243828 showed a significant association with AF in two independent population (GeneID‐north population with 694 AF cases and 710 controls, adjusted *P_‐adj_ *= 6.25 × 10^−3^ with an odds ratio was 0.77, GeneID‐central population with 1106 cases and 1501 controls, *P_‐adj_* = 9.88 × 10^−5^ with an odds ratio was 0.75). The results also showed G allele was significantly associated with lower plasma concentration of myeloperoxidase in general population. We also observed a significant difference of odds ratio between subgroups of hypertension and non‐hypertension. Therefore, our findings identified variant in *MPO* associated with risk of AF and it may give strong evidence to link the inflammation with the incidence of AF.

## 1 INTRODUCTION

Atrial fibrillation (AF) is the most common type of cardiac arrhythmia and characterized by rapid and irregular beating of the atria.^1^ The mechanisms underlying AF are complex and not clear. Myeloperoxidase (MPO) is a crucial regulator of modulating MMP activity and strongly linked to the oxidative stress and inflammation process of remodelling in atria.^2^ Studies showed that plasma MPO level was higher in AF patients than in common controls, and AF patients with high MPO levels showed an increased risk of recurrence after catheter ablation.^3^, ^4^, ^5^, ^6^ Considering the important roles of oxidative stress and inflammation in the initiation and maintenance of AF,^7^ it remains unclear whether MPO acts as a passive marker or as a risk factor in AF.

In this study, we evaluated the association between rs2243828, a variant in promoter region of MPO gene and the risk of AF in two independent case‐control populations totally contained 1800 AF cases and 2211 controls from a Chinese GeneID population.

## 2 METHODOLOGY

### 2.1 Study patients

All samples were from the GeneID database.^8^ The study was approved by Medical Ethical Committee of HUST and conformed to the guidelines set forth by the Declaration of Helsinki. Written informed consent was obtained from all patients. All patients were Han origin according to the medical records.

AF diagnosis was following the ACC/AHA/ESC guidelines.^9^ Patients with other types of cardiac arrhythmias, congenital heart disease, thyroid dysfunction, cardiomyopathies and valvulopathies were excluded. Lone AF was defined as an AF patient that had no CAD, congestive heart failure, hypertension and diabetes.

All patients in the GeneID‐north population mainly enrolled in the hospitals of north of China (Dalian, Liaoning province) and in GeneID‐central population mainly in the hospitals of central of China (Hubei province). All the cases and controls were geographically matched.

Control patients were individuals without any evidence of AF and other types of arrhythmias based on data from ECG or echocardiography.

### 2.2 Genotyping

Genotyping for SNP rs2243828 was carried out using the TaqMan allelic discrimination assay as previously described.^10^ The genotyping results of TaqMan assay were verified by direct Sanger DNA sequencing of 48 patients randomly selected.

### 2.3 Plasma myeloperoxidase measurements by enzyme‐linked immunosorbent assay

To determine whether rs2243828 associated with quantity of myeloperoxidase in plasma, plasma myeloperoxidase concentrations of 177 common health people we measured by ELISA (CUSBIO), and the difference of the mean values (ng/mL) between different genotype carriers were compared using Student's t test.

### 2.4 Statistical analysis

Pearson's 2 × 2 contingency tables chi‐square tests were used to analyse the allelic association, and multivariate logistic regression model was used to adjust the potential confounders. Statistical power analysis was carried out by PS, Power and Simple Size Calculation software.^11^

## 3 RESULTS

### 3.1 Study populations

The demographic and clinical characteristics of the two study population are shown in Table 1. The GeneID‐north population consisted of 694 AF patients (452 were diagnosed as lone AF) and 710 controls, and the GeneID‐central population consisted of 1016 AF patients (355 were diagnosed as lone AF).

**TABLE 1:** Demographic and clinical characteristics of study populations

Characteristics	GeneID‐north population			GeneID‐central population		
	Cases (n = 694)	Controls (n = 710)	*P* ^b^	Cases (n = 1016)	Controls (n = 1501)	*P* ^b^
Age^a^, mean ± SD (years)	60.07 ± 10.85	62.14 ± 9.11	0.01	58.24 ± 9.19	58.62 ± 11.22	0.18
Sex, No.% of females	295 (42.5)	325 (45.8)	0.22	420 (41.33)	706 (47.03)	4.80 × 10^−3^
Hypertension, No.%	207 (29.8)	220 (30.99)	0.60	246 (24.99)	386 (25.72)	0.39
Diabetes, No.%	101 (14.6)	127 (17.9)	0.09	132 (12.99)	170 (11.33)	0.21
Lone AF	452 (65.1)	0	N.A	355 (34.94)	0	N.A
CAD	34	46	0.20	110	181	0.48

Note

Data are shown as mean ± standard deviation (SD) for quantitative variables and per cent (%) for qualitative variables.

^a^ Age at the first diagnosis of the disease in AF case and at enrolment of AF controls.

^b^ *P* values for comparison of means between cases and controls for quantitative variables and Pearson's 2 × 2 contingency tables χ^2^ tests for qualitative variables.

Statistical power analyses showed that our study populations have enough power to detect an association between rs2243828 with AF in GeneID‐north population (78% power) or in GeneID‐central population (100%) with a type I error of 0.05 under the assumptions that OR greater than 1.3 and a minor allelic frequency of 0.16.

### 3.2 G allele of SNP rs2243828 in the promoter region of *MPO* gene confers a significant protective effect to AF in the Chinese Han population

**FIGURE 1:**
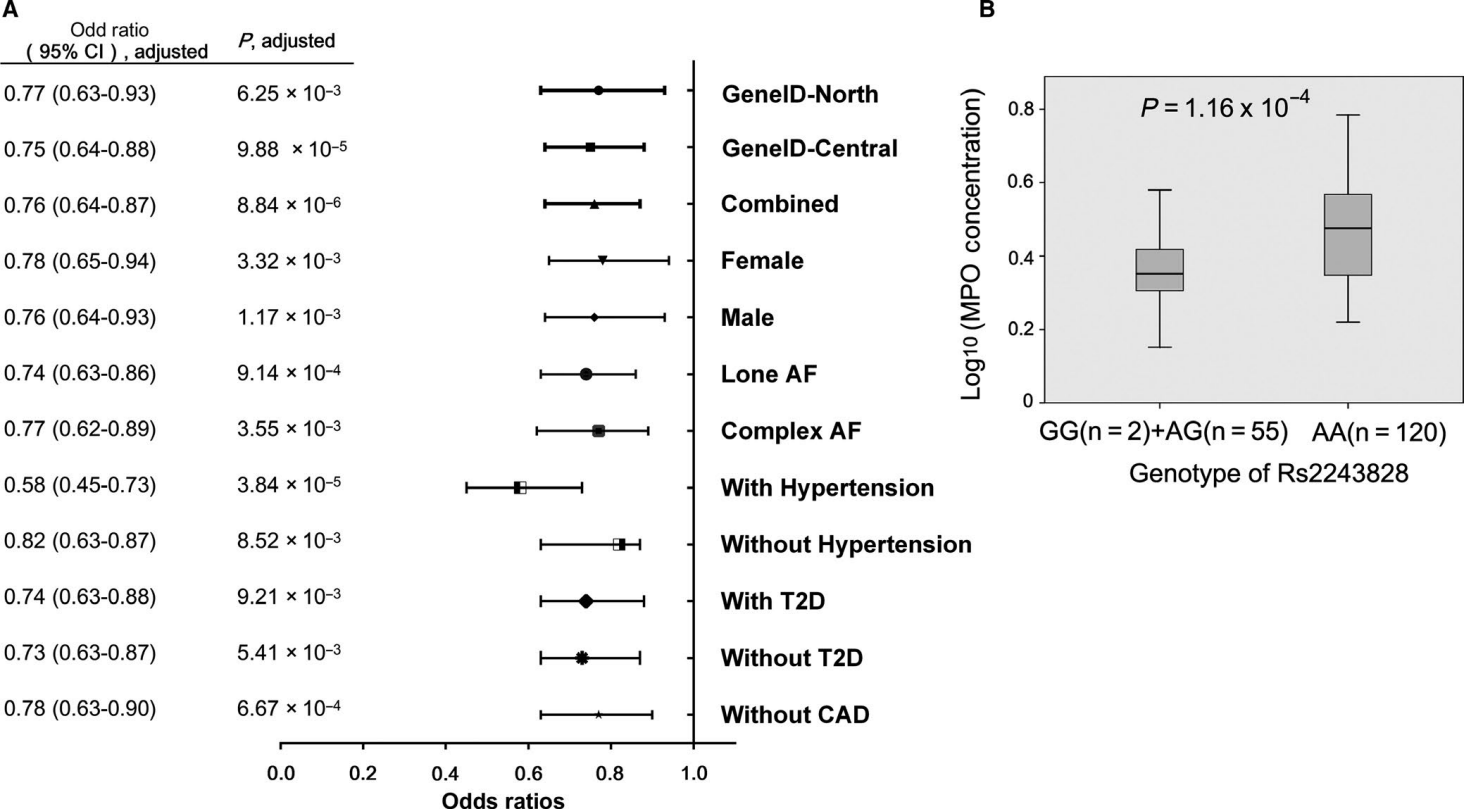
Significant association of SNP rs2243828 with AF and plasma concentration of myeloperoxidase. A, Association between rs2243828 and AF. Adjusted *P* value was obtained using multivariate logistic regression analysis for potential confounders including age, gender, hypertension and diabetes mellitus. B, Difference of plasma concentration of myeloperoxidase between patients with different genotype of rs2243828. Plasma concentration was measured by ELISA

Genotyping data of rs2243828 did not deviate from the Hardy‐Weinberg equilibrium in the controls in both two populations (*P* = 0.66 in GeneID‐north population and 0.81 in GeneID‐central population). In the GeneID‐north population, the frequency of G allele of rs2243828 in 694 AF cases was 0.14 and compared with 0.18 in 710 AF free controls. The G allele conferred significant protective effect of AF with an OR of 0.74 and a *P* value (*P‐obs*) of 7.65 × 10^−3^. After adjusting for potential confounders including age, hypertension, gender and diabetes mellitus, the association remained significant (OR = 0.77 with an adjusted *P* or *P‐adj* = 6.25 × 10^−3^) (Figure 1A).

In GeneID‐central population, the G allele of also significantly associated with AF (*P‐obs* = 7.28 × 10^−5^, *P‐adj* = 9.88 × 10^−5^, OR = 0.75) (Figure 1A). After combining two populations, which totally included 1800 cases and 2211 controls, SNP rs2243828 showed highly significant association with AF (*P‐obs* = 2.42 × 10^−6^, *P‐adj* = 8.84 × 10^−6^, OR = 0.76) (Figure 1A).

The significant association was also observed when the population were divided into subgroups in condition of gender, hypertension, type 2 diabetes, without CAD and lone or complex AF (Figure 1A).

A Breslow‐Day test was used to compare the homogeneity of ORs between different subgroups to analyse whether rs2243828 interacted with risk factors, we observed a significant higher effect in subgroups of hypertension than non‐hypertension (observed OR = 0.55 in patients with hypertension and 0.81 in patients without hypertension, *P* = 3.11 × 10^−4^).

### 3.3 Plasma Myeloperoxidase concentration was lower in G carriers of rs2243828 than in A allele carriers

We measured plasma myeloperoxidase in 177 common health people by ELISA and found that the concentration was lower in 57 G allele carriers (55 AG and 2 GG, 9.42 ± 4.12 ng/mL) than in 120 patients with AA genotype ( 21.20 ± 18.69 ng/mL) (*P* = 1.16 × 10^−4^) (Figure 1B). These results showed that G allele of rs2243828 associated with lower plasma Myeloperoxidase concentration.

## 4 DISCUSSION

A key mechanistic link by which MPO contributes to atrial fibrillation seems to be atrial fibrosis.^2^ MPO is a crucial regulatory switch modulating matrix metalloproteinases (MMPs) activity, and MMPs were key effectors of extracellular matrix (ECM) turnover and remodelling in atrial fibrillation. *Mpo* ^−/−^ mice showed markedly reduced atrial fibrosis and were almost completely protected from the increased vulnerability to atrial fibrillation caused by AngII administration, and both of these effects were dose‐dependently reversed upon restitution of MPO.^2^ Thus, MPO acts as a major downstream mediator of atrial fibrosis and atrial fibrillation.

Our study also showed a larger effect of rs2243828 to AF in patients with hypertension. Hypertension is one of the most important risk factors associated with atrial fibrillation, and the pathophysiological link between hypertension and AF is unclear. Our results indicated that MPO and inflammation may mediate pathophysiological process of hypertension to AF.

In conclusion, we indicated that G allele of rs2243828, which showed related with lower plasma MPO concentration, was associated with reduced AF risk. To the best of our knowledge, this is the first time that the genetic variant in MPO was shown to be associated with a risk of AF in human population. These results show that the inflammation may confer risk in the pathogenesis of AF. Therefore, restriction of inflammation may be a potential scheme for the prevention and treatment of AF.

## CONFLICT OF INTEREST

The authors confirm that there are no conflicts of interest.

## AUTHOR CONTRIBUTION

**Pengyun Wang:** Data curation (lead); Formal analysis (equal); Funding acquisition (equal); Writing‐original draft (equal). **Mian Cheng:** Data curation (equal); Methodology (equal); Resources (equal). **Pengxia Wang:** Data curation (equal); Formal analysis (equal); Methodology (equal). **Liang Xiong:** Methodology (equal); Resources (equal). **Yali Zeng:** Data curation (equal). **Xin Tu:** Conceptualization (equal); Writing‐review & editing (equal). **Rongfeng Zhang:** Data curation (equal); Resources (equal). **Yunlong Xia:** Project administration (equal); Resources (equal). **Gang Wu:** Methodology (equal); Project administration (equal); Resources (equal). **Qing K. Wang:** Conceptualization (equal); Funding acquisition (equal); Project administration (equal); Supervision (equal); Writing‐review & editing (equal). **Xiang Cheng:** Conceptualization (equal); Project administration (equal); Supervision (equal); Writing‐review & editing (equal). **Chengqi Xu:** Conceptualization (equal); Funding acquisition (equal); Project administration (equal); Supervision (equal); Writing‐review & editing (lead).

## Data Availability

The data that support the findings of this study are available from the corresponding author upon reasonable request.
